# Comparison of surgical and conservative treatment outcomes for type a aortic dissection in elderly patients

**DOI:** 10.1186/s13019-018-0814-6

**Published:** 2018-12-18

**Authors:** Takeshi Aoyama, Susumu Kunisawa, Kiyohide Fushimi, Teiji Sawa, Yuichi Imanaka

**Affiliations:** 1 Department of Anesthesiology, Graduate School of Medicine, Kyoto Prefectural University of Medicine, Kyoto, Japan; 2 Department of Healthcare Economics and Quality Management, Graduate School of Medicine, Kyoto University Yoshida Konoe-cho, Sakyo-ku, Kyoto, 606-8501 Japan; 3 Department of Health Policy and Informatics, Graduate School of Medicine, Tokyo Medical and Dental University, Tokyo, Japan; 4 Department of Anesthesiology, Omihachiman Community Medical Center, Shiga, Japan

**Keywords:** Aortic dissection, Elderly, Mortality, Complication, Activities of daily living, Propensity score matching analysis

## Abstract

**Background:**

In recent years, surgical outcomes have improved, and positive reports on surgery for type A aortic dissection (AAD) in the elderly are increasing. However, the difference between surgical and conservative treatments in the elderly remains unclear. Therefore, we conducted this study to determine whether surgery should be performed for Stanford (AAD) in elderly patients.

**Methods:**

Data of patients aged 80 years or older who were hospitalized for AAD from April 2014 to March 2016 were extracted from the Japanese national inpatient database. Outcome measures were all-cause in-hospital death, stroke, acute kidney injury and tracheotomy, and composite adverse events (consisting of all-cause in-hospital death, stroke, acute kidney injury, and tracheotomy), and we compared them between surgical and conservative treatments using propensity score matching.

**Results:**

The study cohort included 3258 patients, with 845 matched pairs (1690 patients) in the propensity score matching. All-cause in-hospital death was significantly lower in the surgical treatment group than in the conservative treatment group before and after matching (15.6% vs. 51.1%, *p* < 0.001; 16.7% vs. 31.6%, *p* < 0.001, respectively); however, there was no significant difference in composite adverse events after matching (36.0%, conservative vs. 37.2%, surgical; *p* = 0.65), and adjusted odds ratio was 1.06 and 95% confidence interval was 0.86–1.29 (*p* = 0.61) with reference to conservative treatment.

**Conclusions:**

All-cause in-hospital death among elderly patients with AAD was significantly lower in patients treated surgically than in those undergoing conservative treatment. However, there was no significant difference between the two groups in the event-free survival, which is important for the elderly. These findings may be used in the consideration of treatment course for elderly patients with AAD.

**Electronic supplementary material:**

The online version of this article (10.1186/s13019-018-0814-6) contains supplementary material, which is available to authorized users.

## Background

The ageing of populations is presenting new challenges to developed countries worldwide. In 2015, 26.7% of the Japanese population was over 65 years of age, and 8.0% of the population was over 80. The fraction of the Japanese population aged 80 or older is estimated to reach 10% by 2025 [1]. The opportunity for elderly patients with fatal diseases to be transported is increasing, and type A aortic dissection (AAD) is no exception. However, whether emergency surgical intervention should be performed in elderly patients is controversial because of the high postoperative mortality and complication rates associated with the extreme invasiveness of this procedure, and age of ≥80 years is a significant risk factor associated with postoperative short-term mortality rate [2–6]. As previously reported by Hata et al. [4], surgery may not necessarily lead to a good outcome for patients and their family, as the elderly may become bedridden or depressed after surgery. However, because AAD has a high mortality rate (42.9–63.6%) without surgery [7–11], whether surgery should be performed for an elderly patient is an ethically difficult decision for surgeons. Clarifying the difference between the outcomes including mortality as well as complications and physical functions of conservative and surgical management of AAD in the elderly may help in therapeutic decision making in patients with AAD admitted to the emergency room.

Although retrospective cohort studies have compared conservative and surgical management of AAD, the patient cohorts of these studies were not adjusted for background. Thus, many inoperable patients with severe conditions may have been included in the non-surgical treatment group, making comparison of the two groups by randomization difficult. Therefore, this study compares the conservative and surgical management of patients with AAD aged > 80 years after adjusting for severity by propensity scores using data from the national inpatient database in Japan.

## Methods

### Data collection

We performed a retrospective cohort study using the Japanese Diagnosis Procedure Combination (DPC) database. The DPC system was introduced in Japan in 2003 as a comprehensive daily evaluation system for acute-phase hospital medical care. In 2016, DPC-participating hospitals accounted for approximately 80% of acute-phase beds in Japan. Clinical information and other data for patients were registered in the DPC database and classified by a 14-digit DPC code. The system holds the following clinical information: patient demographics, diagnoses, comorbidities and complications [registered using the International Classification of Disease, 10th revision (ICD10)], drugs and devices used, procedures performed, healthcare costs and status at admission and discharge.

### Patient selection

Data were extracted from the DPC database between April 2014 and March 2016. First, we identified patients aged 80 years or older with acute aortic dissection (ICD10 code, I71.0) recorded as either the “main diagnosis,” “admission-precipitating diagnosis,” or “most resource-consuming diagnosis” and identified patients with dissection in the ascending aorta with “Stanford type A,” “DeBakey I,” or “DeBakey II” as the disease name. We excluded patients who had planned admission and unknown admission pathway. The final cohort was divided into the conservative and surgical treatment groups.

### Variables and outcomes

We matched propensity scores between the surgical and conservative treatment groups. Propensity scores were calculated using a logistic regression model with the following variables: age, sex, body mass index (divided into the four following groups: < 18.5, 18.5–25.0, > 25.0, and data missing), admission pathway (home, other hospital, or clinic and nursing-care facility), ambulance use, hypertension, diabetes mellitus, chronic obstructive pulmonary disease (COPD), chronic kidney disease, ischemic heart disease, chronic liver disease, cancer, dementia, cardiopulmonary arrest, cardiac tamponade, shock, coronary malperfusion, limb ischemia, aortic insufficiency, hospital volume [low (< 10 cases), middle (10–20 cases), and high (>20cases)] and consciousness level upon admission (Japan coma scale: 0, alert; 1–3, delirious; 10–30, somnolent and 100–300, coma). Outcome measures were all-cause in-hospital death, stroke, and acute kidney injury (AKI) requiring renal replacement therapy (RRT), tracheotomy, composite adverse events (all-cause in-hospital death, stroke, AKI, and tracheotomy), length of hospital stay, length of intensive care unit (ICU) stay, inpatient costs, home discharge rate, and activities of daily living (ADL) of surviving patients at discharge. ADL was evaluated using the Barthel index (BI), which assesses the ADL using a 100-point scale comprising the following 10 items: “Bowels,” “Bladder,” “Grooming,” “Toilet use,” “Feeding,” “Transfer,” “Mobility,” “Dressing,” “Stairs,” and “Bathing,” and we set the BI cut-off at 60 points, which indicates having functional dependency. [12, 13].

### Statistical analysis

Summary statistics are presented as frequencies and percentages for categorical variables and median and interquartile ranges for continuous variables (with non-normal distribution in the Shapiro–Wilk test). Data were compared using the Mann–Whitney U test for continuous variables and the chi-squared test for categorical variables before matching; after matching, the Wilcoxon signed rank and McNemar tests were performed. We performed one-to-one pair matching using nearest neighbor matching without replacement within 0.2 standard deviations of the logit of the propensity score as caliper width [12]. Covariate balance between the two groups was checked depending on whether the absolute standardized difference was < 10% [13]. Adjusted odds ratios (AOR) for the primary outcome were estimated using multivariable logistic regression in all patients aged > 80 years to investigate the influence of the type of surgery. As a subgroup analysis, the status of surviving patients at discharge in both the groups was compared before after matching. The robustness of the result was confirmed using the following method as sensitivity analysis for the BMI missing value (749 cases): list-wise deletion method (excluded the BMI missing data), median imputation for BMI missing data and multiple imputation for BMI missing data. All analyses were performed using the R statistical software (version 3.5.1; R Foundation for Statistical Computing, Vienna, Austria), and the packages used for analyses were “Matching” for propensity score matching and “mice” for missing value complement. *P* < 0.05 was considered significant, and hypothesis tests were two sided. This study was performed with the approval of the Ethics Committee, Kyoto University Graduate School and Faculty of Medicine (Approval number: R0135).

## Results

**Fig. 1:**
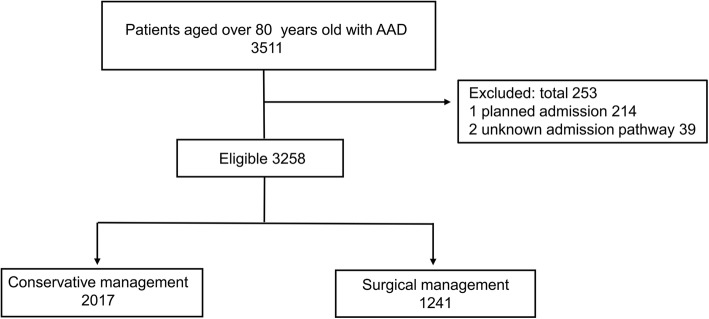
Patient selection

**Fig. 2:**
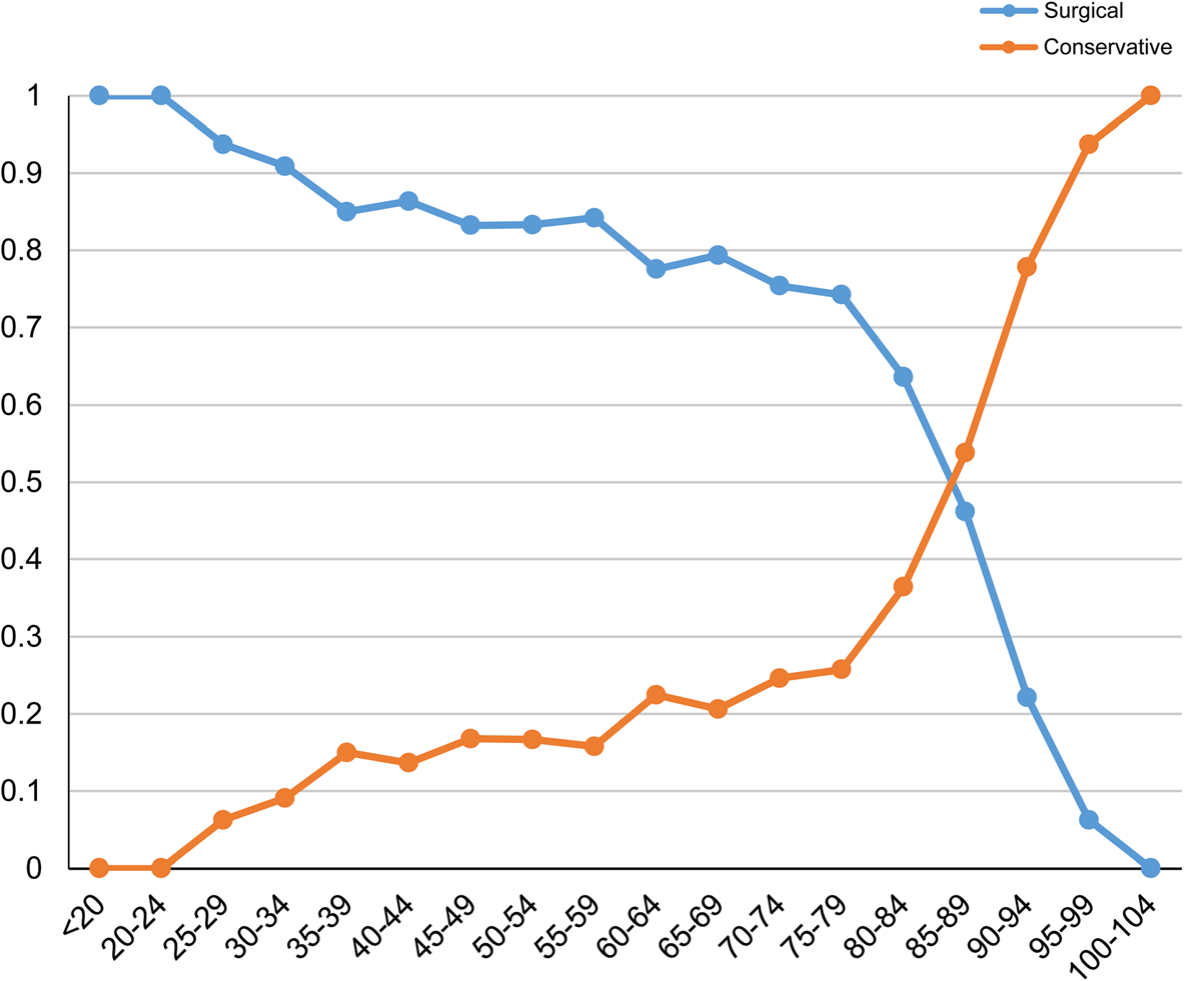
Management of AAD based on the age group (5-year increments)

The process used to select eligible patients is shown in Fig. 1. The total patient cohort included 3258 patients (surgical treatment group, *n* = 1241; conservative treatment group, *n* = 2017) who were hospitalized in a total of 634 hospitals. The study had 845 matched pairs (1690 patients) in the propensity score matching. As shown in Fig. 2, the fraction of patients treated conservatively increased with increasing age, with a steep increase for patients over 80 years of age. Patient background and clinical findings before and after matching are compared in Table 1. Before matching, the age of the conservative treatment group was significantly higher than that of the surgical treatment group [86.0 years (82.0–89.0) vs. 83.0 years (81.0–86.0), respectively]. The rate of patients transferred from other hospitals was significantly higher for the surgical treatment group, whereas that of patients from nursing-care facilities was significantly higher for the conservative treatment group. Dementia was frequently observed in the conservative treatment group (9.5% vs. 6.4%). The surgical treatment group had considerably more patients with a good level of consciousness upon admission. The rate of cardiopulmonary arrest was also higher in the conservative treatment group (10.0% vs. 0.4%), whereas shock and aortic insufficiency were higher in the surgical treatment group. Surgical management was often selected in high volume hospitals. All baseline characteristics between the groups after matching remained balanced, with the absolute standardized effect size < 10%.

**Table 1:** Baseline characteristics of all patients over 80 years of age vs. propensity-matched patients

	All patients over 80 years of age			Propensity-matched patients		
Variables	Conservative	Surgical	Absolute Standardised Difference,%	Conservative	Surgical	Absolute Standardised Difference,%
	*n* = 2017	*n* = 1241		*n* = 845	*n* = 845	
Age, years; median [IQR]	86.0 [83.0–89.0]	83.0 [81.0–86.0]	88.2	84.0 [81.0–87.0]	84.0 [82.0–86.0]	1.9
Gender (male), n (%)	524 (26.0)	315 (25.4)	1.4	234 (27.7)	227 (26.9)	1.6
BMI, n(%)						
< 18.5	307 (15.2)	172 (13.9)	3.9	129 (15.3)	121 (14.3)	0.3
18.5–25.0	867 (43.0)	786 (63.3)	42.2	508 (60.1)	525 (62.1)	4.1
>25.0	188 (9.3)	189 (15.2)	16.4	126 (14.9)	113 (13.4)	4.9
BMI data missing	655 (32.5)	94 (7.6)	94.1	82 (9.7)	86 (10.2)	1.6
Ambulance use, n (%)	1640 (81.3)	1038 (83.6)	6.3	680 (80.5)	686 (81.2)	0.3
Admission pathway, n (%)						
Home	1598 (79.2)	975 (78.6)	1.6	708 (83.8)	697 (82.5)	4.0
Other hospital or clinic	172 (8.5)	244 (19.7)	28.0	118 (14.0)	128 (15.1)	4.0
Nursing-care facility	247 (12.2)	22 (1.8)	79.3	19 (2.2)	20 (2.4)	0.8
Hypertension, n (%)	867 (43.0)	695 (56.0)	26.2	476 (56.3)	458 (54.2)	5.0
Diabetes mellitus, n (%)	88 (4.4)	114 (9.2)	16.7	67 (7.9)	65 (7.7)	0.9
COPD, n (%)	68 (3.4)	45 (3.6)	1.4	29 (3.4)	35 (4.1)	3.0
Chronic kidney disease, n (%)	63 (3.1)	54 (4.4)	6.0	35 (4.1)	35 (4.1)	1.2
Ischemic heart disease, n (%)	162 (8.0)	163 (13.1)	15.1	107 (12.7)	109 (12.9)	0.4
Chronic Liver disease, n (%)	32 (1.6)	42 (3.4)	9.9	23 (2.7)	22 (2.6)	0.7
Cancer, n (%)	91 (4.5)	35 (2.8)	10.2	30 (3.6)	32 (3.8)	0.6
Dementia, n (%)	191 (9.5)	80 (6.4)	12.3	70 (8.3)	55 (6.5)	7.2
JCS at admission, n (%)						
(alert)	937 (46.5)	890 (71.7)	56.1	555 (65.7)	562 (66.5)	1.0
1–3 (delirious)	410 (20.3)	206 (16.6)	10.0	180 (21.3)	161 (19.1)	4.8
10–30 (somnolent)	161 (8.0)	57 (4.6)	16.2	48 (5.7)	45 (5.3)	0.0
100–300 (coma)	509 (25.2)	88 (7.1)	70.7	62 (7.3)	77 (9.1)	4.9
Cardiopulmonary arrest on arrival,n(%)	201 (10.0)	5 (0.4)	150.9	1 (0.1)	5 (0.6)	4.6
Cardiac tamponade, n (%)	321 (15.9)	178 (14.3)	4.5	109 (12.9)	124 (14.7)	1.5
Shock, n (%)	153 (7.6)	226 (18.2)	27.5	93 (11.0)	96 (11.4)	3.6
Coronary malperfusion, n (%)	50 (2.5)	24 (1.9)	4.0	93 (11.0)	96 (11.4)	4.2
Limb ischemia, n (%)	99 (4.9)	71 (5.7)	3.5	36 (4.3)	46 (5.4)	3.0
Aortic insufficiency, n (%)	41 (2.0)	143 (11.5)	29.7	33 (3.9)	50 (5.9)	7.5
Hospital volume, n (%)						
Low	1302 (64.6)	571 (46.0)	37.2	465 (55.0)	452 (53.5)	4.0
Middle	548 (27.2)	448 (36.1)	18.6	285 (33.7)	306 (36.2)	5.4
High	167 (8.3)	222 (17.9)	25.1	95 (11.2)	87 (10.3)	1.9

Outcomes are shown in Tables 2 and 3. All-cause in-hospital death was higher in the conservative treatment group even after matching (31.6% vs. 16.7%) (AOR, 0.42; 95% CI, 0.33–0.54; *p* < 0.001), but complications and tracheotomy during hospitalization were high in the surgical treatment group both before and after matching: stroke (3.5% vs. 17.2%)/(4.5% vs. 17.5%), acute kidney injury (1.0% vs. 8.8%)/(1.7% vs. 8.6%), and tracheostomy (0.8% vs. 8.7%)/(1.1% vs. 9.2%). Composite adverse events occurred in 54.0 and 36.6% in conservative and surgical treatment groups, respectively, before matching but occurred in 36.0 and 37.2%, respectively, after matching, which was not significant.

**Table 2:** Comparison of postoperative outcomes between conservative and surgical treatment

		All patients > 80 years of age			Propensity-matched patients		
Outcome		Conservative	Surgical	*p*	Conservative	Surgical	*p*
		(*n* = 2017)	(*n* = 1241)		(*n* = 845)	(*n* = 845)	
All-cause in-hospital death		1031(51.1)	193 (15.6)	< 0.001	267 (31.6)	141 (16.7)	< 0.001
Stroke, n (%)		71 (3.5)	213 (17.2)	< 0.001	38 (4.5)	148 (17.5)	< 0.001
AKI, n (%)		21 (1.0)	109 (8.8)	< 0.001	14 (1.7)	73 (8.6)	< 0.001
Tracheotomy, n (%)		16 (0.8)	108 (8.7)	< 0.001	9 (1.1)	78 (9.2)	< 0.001
Composite adverse events		1090 (54.0)	454 (36.6)	< 0.001	304 (36.0)	314 (37.2)	0.65
Length of hospital stay, days	Median [IQR]	7.0 [1.0–26.0]	31.0 [20.0–46.0]	< 0.001	17.0 [3.0–31.0]	31.0 [21.0–48.0]	< 0.001
Length of ICU stay,days	Median [IQR]	1.0 [0.0–4.0]	7.0 [4.0 12.0]	< 0.001	2.0 [0.0–6.0]	7.0 [4.0–12.0]	< 0.001
Inpatient cost, (US $)	Median [IQR]	4200 [1400–10,200]	36,600 [30300–45,400]	< 0.001	7200 [2700–12,100]	36,400 [30300–45,900]	< 0.001

**Table 3:** Adjusted odds ratio for postoperative outcomes with reference to conservative treatment in the propensity matched patients

Propensity matched patients			
Outcome	Adjusted odds ratio	95% CI	*P*
All-cause in-hospital death	0.42	0.33–0.54	< 0.001
Stroke	4.44	3.02–6.51	< 0.001
AKI	5.92	3.21–10.91	< 0.001
Tracheotomy	12.50	5.44–28.71	< 0.001
Composite adverse events	1.06	0.86–1.29	0.61

The hospital length of stay and ICU length of stay were significantly longer in the surgical treatment group before/after matching, although the difference was lower after matching. The medical expenses were also significantly higher in the surgical treatment group than in the conservative treatment group before/after matching [$4100 (1400–10,200) vs. $36,600 (30,300–45,400); *p* < 0.001]/[$7200 (2700–12,100) vs. $36,400 (30,300–45,900); *p* < 0.001].

The results of functional status at discharge of surviving patients are shown in Table 4. The home discharge rate was significantly higher for the conservative treatment group both before and after matching (52.8% vs. 42.8%). BI score was significantly higher and patients whose BI was < 60 points was significantly lower in the surgical treatment group, but these differences became insignificant after matching.

**Table 4:** Functional status at discharge for survivors

	All patients > 80 years of age			Propensity matched patients		
	Conservative	Surgical	*p*	Conservative	Surgical	*p*
	*n* = 986/2017	*n* = 1048/1241		*n* = 578/845	*n* = 704/845	
BI score, mean (SD)	50.9 (40.8)	64.6 (37.6)	<.001	59.1 (40.4)	63.6 (37.3)	0.06
BI < 60	442 (54.4)	343 (38.4)	<.001	219 (45.4)	239 (40.3)	0.1
Discharge to home	442 (44.8)	452 (43.1)	0.45	305 (52.8)	301 (42.8)	<.001

The AOR for postoperative outcomes by each type of surgeries is shown in Table 5. As the procedure of the surgery became more complicated, AOR for all-cause in-hospital death and composite events tended to increase. The AOR for postoperative complications and tracheotomy was the highest in the total arch replacement.

**Table 5:** Adjusted odds ratio for postoperative outcomes in the AAD major surgeries with reference to conservative treatment in all patients > 80 years of age

	All cause death adjusted odds ratio (95%CI, *P* value)	Stroke adjusted odds ratio (95%CI, *P* value)	Acute kidney injury adjusted odds ratio (95%CI, *P* value)	Tracheotomy adjusted odds ratio (95%CI, *P* value)	Composite adverse events adjusted odds ratio (95%CI, *P* value)
Ascending aortic replacement or hemiarch replacement,	0.29 (0.23–0.38,< 0.001)	5.60 (3.94–8.05,< 0.001)	6.85 (3.97–12.3, < 0.001)	12.9 (7.00–25.6,< 0.001)	0.86 (0.70–1.05, 0.14)
Total arch replacement	0.34 (0.24–0.48,< 0.001)	6.42 (4.23–9.78,< 0.001)	8.00 (4.27–15.36, < 0.001)	16.5 (8.3–34.3, < 0.001)	1.08 (0.59–1.42, 0.57)
Aortic root replacement	1.67 (0.83–3.33,< 0.001)	5.81 (2.41–12.9,< 0.001)	6.40 (1.69–19.54, < 0.002)	7.66 (1.61–26.8, 0.003)	1.92 (0.98–3.79, 0.06)

Multiple sensitivity analyses about missing data showed similar point estimates and 95% CIs (See Additional file 1).

## Discussion

Advances in minimally invasive surgery and the advent of aortic aneurysm stent grafts and transcatheter aortic valve implantation provide more treatment options for previously difficult-to-treat cardiovascular conditions in high-risk or elderly patients. In contrast, although there are some reports of endovascular treatment for AAD [14, 15], it still requires surgery involving sternotomy and cardiopulmonary bypass under hypothermia. Because such treatment imposes a tremendous burden on elderly patients, the establishment of AAD treatment standards for elderly patients has been controversial. However, the surgical outcomes of these patients are improving because of advancements in surgical techniques, cardiopulmonary bypass and postoperative management. Recent reports of AAD surgery in elderly patients are positive [16–18]. In a meta-analysis of elderly patients with AAD (> 80 years of age), Biancari et al. compared immediate postoperative mortality among patients treated surgically and those treated with conservative therapy using Pool analysis of data reported in two previous studies [13]. Although this study cohort was small, their analysis indicates more favorable outcomes for patients treated surgically (postoperative mortality rate: surgical treatment, 25.2%; non-surgical treatment, 59.0%). Furthermore, postoperative mortality of AAD surgery in the elderly in some reports from Japan is generally low (3.7–13.6%) and favorable, although their age definition of ‘elderly’ varies from 70 to 80 years of age [16–22]. Despite these favorable reports, our survey indicates that only half of the elderly patients with AAD (80 years or older) are treated surgically.

We observed a lower in-hospital mortality rate in patients undergoing surgery than in those receiving conservative treatment (15.6% /16.7%; before/after matching), the risk difference was 14.9% even after matching. These results certainly indicate that surgery is a good treatment option; however, on the other hand, the percentage of patients who could be discharged to home after surgery was approximately 40, and 40% of the patients in the surgical treatment group had BI < 60 and required a lot of assistance. These results support the observation by Hata et al. that AAD surgery poses issues including the risk of being bedridden and/or depressed and a burden on the family [3]. In addition, stroke, acute kidney injury and tracheostomy during hospitalization were significantly higher in the surgical treatment group than in the conservative treatment group, so there was no significant difference in the primary outcome after matching between the groups. Once these complications and events occur in the elderly, there is a high possibility that irreversible function decline will occur in them than in younger patients. Further, the hospital length of stay, ICU length of stay and inpatient costs were higher in the surgical treatment group. However there was no difference in the functional status between the two groups, that is, low-grade stroke or AKI do not necessarily lead to functional decline. These results make it difficult for the elderly with AAD to select a treatment option (Fig. 2).

Extremely positive outcomes have been reported for elderly patients undergoing hemiarch replacement and a short-time/less-invasive ascending aortic replacement for entry closure [16, 21], and favorable outcomes with a good QOL assessment after AAD surgery have been reported for elderly patients with use of 36-Item Short Form Health Survey (SF36) [23, 24]. Our results also show favorable results with ascending aortic or hemiarch replacement. Conversely, Ozaki and colleagues recently proposed medical treatment as an option for AAD, reporting good results with medical treatment for AAD in a small cohort of elderly patients [25]. Our results also show favorable results with ascending aortic or hemiarch replacement, but complex procedures such as root surgery may be worse than conservative therapy.

Few reports have compared conservative and surgical treatment of the elderly, and the results of this study may be useful for choosing treatment plans in patients with AAD. The strengths of this study are that we examined an unprecedented number of AAD cases in the elderly and are the first to compare surgical and conservative treatments using propensity scores and that we compared short-term mortality as well as event-free survival rate and functional status at discharge, which is particularly important in the elderly. However, this study also has some limitations. Because the study cohort includes a very specific population over 80 years of age, the results cannot be extrapolated to different populations. The retrospective design of this study has inherent limitations, especially selection bias and dependence on historical records. Other limitations include a lack of clinical information, including preoperative vital signs, laboratory data/diagnostic imaging, specific anaesthesia method/surgical technique, and postoperative management. Other underlying diseases may not have been identified because of the extraction of comorbidity or findings on admission from registered disease names. Propensity scores may have been influenced by the presence of unmeasured factors. Because our study was short term, the results do not reflect subsequent death after discharge or the later need for surgery. Thus, our results do not address the mid- or long-term outcomes after discharge.

## Conclusions

Our results indicate that AAD surgery is a feasible treatment option for the elderly and has a significantly lower hospital mortality rate than does conservative treatment, even in patients over 80 years of age. Despite these findings, many elderly patients with AAD are not treated surgically. This may be due to postoperative complications and poor condition at discharge. With the growth of older populations in Japan and other countries worldwide, postoperative outcomes of the elderly will be an increasingly important issue, and increase in event-free survival rate will support the choice of AAD surgery for the elderly patients.

## Additional file

Additional file 1: Additional table. Sensitivity analysis for BMI missing data (DOCX 19 kb)

## Abbreviations

AAD: Type A aortic dissection
ADL: Activities of daily living
AKI: Acute kidney injury
AOR: Adjusted odds ratio
BI: Barthel index
COPD: Chronic obstructive pulmonary disease
DPC: Diagnosis procedure combination
ICU: Intensive care unit
RRT: Renal Replacement therapy
