# Supplementary material for: Comparison of surgical and conservative treatment outcomes for type a aortic dissection in elderly patients
Source: J Cardiothorac Surg. 2018 Dec 18;13:129. doi: 10.1186/s13019-018-0814-6 (PMC6299624; doi:10.1186/s13019-018-0814-6)
Supplement: Supplementary file 1 — Additional table. Sensitivity analysis for BMI missing data (DOCX 19 kb) [file 13019_2018_814_MOESM1_ESM.docx]

Additional table. Sensitivity analysis for BMI missing data

|  | **Propensity matching (List wise)** | | |
| --- | --- | --- | --- |
| **Outcome** | **Adjusted odds ratio** | **95% CI** | **P** |
| All-cause in-hospital death | 0.34 | 0.26–0.45 | <0.001 |
| Stroke | 4.28 | 2.85–6.41 | <0.001 |
| Acute kidney injury | 5.64 | 2.97–10.70 | <0.001 |
| Tracheotomy | 8.25 | 3.96–17.18 | <0.001 |
| Composite adverse events | 0.98 | 0.75–1.15 | 0.490 |
|  |  |  |  |
|  |  |  |  |
|  | **Propensity matching (Median imputation)** | | |
| **Outcome** | **Adjusted odds ratio** | **95% CI** | **P** |
| All-cause in-hospital death | 0.37 | 0.29–0.46 | <0.001 |
| Stroke | 5.48 | 3.63–8.26 | <0.001 |
| Acute kidney injury | 4.73 | 2.71–8.26 | <0.001 |
| Tracheotomy | 9.00 | 4.50–17.92 | <0.001 |
| Composite adverse events | 0.91 | 0.75–1.10 | 0.300 |
|  |  |  |  |
|  |  |  |  |
|  | **Propensity matching (Multiple imputation)** | | |
| **Outcome** | **Adjusted odds ratio** | **95% CI** | **P** |
| All-cause in-hospital death | 0.38 | 0.30–0.48 | <0.001 |
| Stroke | 4.34 | 3.00–6.27 | <0.001 |
| Acute kidney injury | 5.31 | 2.93–9.60 | <0.001 |
| Tracheotomy | 9.00 | 4.52–17.80 | <0.001 |
| Composite adverse events | 0.92 | 0.76–1.11 | 0.400 |
